# Learning Natural Categories: Effects of Interleaving Practice in Children and Young Adults

**DOI:** 10.3390/jintelligence13090107

**Published:** 2025-08-25

**Authors:** Xiaoxiao Dong, Xiaoxiao He, Lingyu Fang, Qiang Xing, Rongxia Ren

**Affiliations:** 1 Department of Education, Guangzhou University, Guangzhou 510006, China; 1112308004@e.gzhu.edu.cn (X.D.); 2112408033@e.gzhu.edu.cn (X.H.); 2112408113@e.gzhu.edu.cn (L.F.); 2 School of Education Sciences, Yan’an University, Yan’an 716000, China; ren@yau.edu.cn

**Keywords:** presentation sequence, natural category learning, category learning judgments

## Abstract

While interleaved learning has been shown to enhance young adults’ acquisition of confusable natural categories, its effects on children’s natural category learning remain underexplored. The present study investigated the effects of study schedule (interleaving vs. blocking) on both categorization accuracy and the accuracy of metacognitive judgments during the learning of natural rock categories, comparing children and young adults. In Experiment 1, participants studied under blocked or interleaved conditions and subsequently provided global judgments of their learning. In Experiment 2, we employed a self-paced learning paradigm that required learners to regulate their own study time. Additionally, participants made item-by-item judgments of their learning during the study phase. Across both experiments, we found that interleaved learning significantly improved categorization accuracy, with young adults benefiting more than children. Regarding metacognitive monitoring, interleaving reduced overconfidence in children but led to underconfidence in young adults, as reflected in both global and item-level judgments. These findings suggest that the benefits of interleaved learning for category performance and metacognitive monitoring vary with age, highlighting age-related differences in the effectiveness of interleaved learning.

## 1. Introduction

Imagine you are a fourth-grade science teacher preparing to introduce your students to different types of rocks. Should you present one type of rock at a time, or alternate between different types? Despite the widespread use of blocked formats—where the same category is presented consecutively—in textbooks (e.g., 91% of examples in middle school math textbooks; Rohrer et al. (2020)), this approach may not be optimal. A growing body of research supports interleaved learning as a more effective approach, especially for young adults (Birnbaum et al. 2013; Do and Thomas 2023; Kornell and Bjork 2008; Kang and Pashler 2012; Yan and Sana 2021; Zulkiply and Burt 2013). Blocked learning involves presenting examples from the same category in succession (e.g., A1 A2 A3 B1 B2 B3 C1 C2 C3), while interleaved learning presents examples from different categories in an alternating order (e.g., A1 B1 C1 A2 B2 C2 A3 B3 C3).

Recent studies have demonstrated that interleaved learning enhances young adults’ ability to classify complex natural categories such as types of rocks (Do and Thomas 2023). Yet, it remains unclear whether the same benefit extends to children. Natural rock categories often involve multiple perceptual dimensions, some of which are subtle and difficult to articulate (Meagher et al. 2022). While prior studies suggest that interleaving can benefit children’s learning (e.g., Vlach and DeBrock 2017; Vlach and Johnson 2013), these studies typically used simplified or artificial stimuli, rather than complex natural categories like rocks. Moreover, most existing research on interleaving focuses primarily on classification accuracy. Less is known about whether interleaving supports metacognitive accuracy—that is, learners’ ability to judge their own category learning performance accurately. This study explores two main questions: (1) Does the sequence in which examples are presented—blocked or interleaved—affect children’s and young adults’ classification accuracy? (2) Does it also influence the accuracy of their learning judgments? In the following section, we briefly summarize relevant prior findings and present the rationale for the current study.

### 1.1. The Mechanism of Interleaving Effect on Category Learning

A substantial body of research has shown that interleaved learning benefits the young adults’ acquisition of highly similar—or confusable—categories (Abel et al. 2024; Brunmair and Richter 2019). Researchers have suggested that interleaved learning environments support category learning by encouraging learners to compare and contrast features across categories—a process known as the discriminative-contrast hypothesis (Kornell and Bjork 2008). Building on this idea, Carvalho and Goldstone (2014) found that interleaving was particularly effective when the categories being learned were highly similar, whereas blocked learning showed advantages for categories with low within-category similarity. To explain this, they proposed the Sequential Attention Theory, which extends the discriminative-contrast hypothesis by emphasizing the role of attentional shifts triggered by the sequencing of examples. According to this theory, encountering items from different categories in succession helps direct learners’ attention to diagnostic, contrasting features. In contrast, when items from the same category are presented consecutively, learners are more likely to attend to shared features.

Although many studies have attributed the benefits of interleaved learning to enhanced contrast and discrimination between categories, researchers have also found advantages of interleaving even when such contrastive processing is not required (Foster et al. 2019). Pan et al. (2025) proposed that multiple cognitive mechanisms can underlie interleaved learning, and that the dominant mechanism may vary depending on the nature of the learning material. One alternative explanation is the study-phase retrieval hypothesis, which emphasizes the role of memory retrieval processes. According to this view, interleaving enhances long-term retention by increasing the spacing between repeated exposures to the same category, thereby promoting more effortful and deeper retrieval at each re-encounter (Foster et al. 2019; Vlach and Kalish 2014).

Researchers have found that the benefits of interleaved learning on learning emerge gradually during development, likely in parallel with improvements in memory retrieval and encoding abilities. According to Vlach and Johnson (2013), 16-month-old infants performed better in blocked conditions, which they attributed to difficulties in retrieving exemplars presented earlier in interleaved sequences. Based on this, they argued that interleaving imposes high cognitive demands that may exceed infants’ retrieval capacity. In contrast, Schwab and Lew-Williams (2016) found that 2-year-old children benefited from interleaved learning during word learning tasks, suggesting developmental progression in retrieval abilities. Similarly, Vlach and DeBrock (2017) showed that preschool-aged children (around 3 years old) retained word mappings better when exposed to interleaved learning than to blocked learning. This enhancement has been linked to more robust encoding and retrieval processes fostered by interleaved practice (Vlach and DeBrock 2017).

To sum up, the advantages of interleaved learning can begin to appear during childhood, as memory retrieval abilities develop—particularly in situations where retrieval is the main underlying mechanism (e.g., Vlach and Johnson 2013; Vlach and Kalish 2014). However, when interleaving involves highly similar or easily confusable categories, its effectiveness is more likely driven by attentional processes that help learners focus on the most relevant distinguishing features (Carvalho and Goldstone 2014). Alternating between categories enhances this contrastive processing and encourages learners to ignore irrelevant or non-diagnostic features (Nemeth et al. 2021). Yet, such attentional flexibility is still developing in children, especially around the age of 9. Compared to adults, children aged 7–10 are less effective at focusing on relevant information while ignoring irrelevant or distracting details (Frank et al. 2021). This difficulty is further compounded when learning natural rock categories, which are often defined by multiple perceptual dimensions—many of which are subtle, complex, or difficult to verbalize (Meagher et al. 2022). Although interleaved learning has been shown to support young adults in learning natural rock categories, it remains unclear whether children as young as 9 years old benefit in the same way. Therefore, one of the key aims of the present study is to examine whether the effects of interleaved learning on natural category acquisition differ between children and young adults.

### 1.2. The Effect of Interleaving on Category Learning Judgments

Interleaved learning affects not only cognitive performance (e.g., classification accuracy), but also metacognitive aspects of learning. One commonly used indicator of metacognition in category learning is category learning judgments (CLJs), which refer to learners’ predictions about their ability to correctly classify new examples that were not encountered during the learning phase but belong to the same categories they studied. Specifically, participants are asked to estimate how likely they are to accurately classify such new items into the appropriate categories. Previous research has shown that participants tend to make higher judgment when studying with blocked sequences compared to interleaved ones (Janssen et al. 2023). However, other findings suggest a more nuanced picture. For example, Wahlheim et al. (2011) found no significant difference in judgment between the interleaved and blocked conditions.

Importantly, the magnitude of judgment does not necessarily reflect their accuracy. One way to assess judgment bias is by examining the discrepancy between participants’ predicted performance (i.e., judgment) and their actual classification performance (Hartwig and Dunlosky 2017). Bias is typically calculated by subtracting actual performance from the predicted judgment. Positive values indicate overestimation, whereas negative values indicate underestimation. Whereas adults typically underestimate their learning performance (O’Leary and Sloutsky 2019), children between the ages of 8 and 11 are generally more optimistic, tending to overestimate how well they have learned (van Loon et al. 2017).

Although interleaving has shown promise in improving young adults’ performance in natural rock category learning, its effect on their judgment accuracy remains unclear. Compared to blocked practice, interleaving can enhance classification performance by promoting discriminative contrast when learning confusable categories (e.g., Abel et al. 2024; Birnbaum et al. 2013; Kornell and Bjork 2008; Kang and Pashler 2012; Wahlheim et al. 2011). Previous research suggests that young adults tend to underestimate their own perceptual learning performance (O’Leary and Sloutsky 2019), possibly due to their failure to utilize valid cues during learning. By highlighting distinctions between categories, interleaving may help learners become more aware of their own level of mastery. This increased awareness might encourage them to draw on meaningful cues from the learning process when making judgments, thereby promoting young people to be able to accurately monitor their performance.

However, an alternative possibility is that interleaving could increase judgment bias in young adults. Young adults often use perceived mental effort as a cue for learning, such that tasks experienced as more effortful are judged as less well learned (Koriat et al. 2006). Interleaved practice has been found to induce greater cognitive effort (Kirk-Johnson et al. 2019), which may lead to a subjective sense of disfluency. Learners often misinterpret fluency as a signal of successful learning, and disfluency as a sign of failure (Koriat 1997). Thus, the disfluency experienced during interleaved learning might further contribute to young adults’ tendency to underestimate their performance. In sum, it remains an open question whether interleaving ultimately improves or impairs young adults’ judgment accuracy.

Children often overestimate their learning performance, possibly because they have not yet developed the ability to accurately interpret accessibility-based cues—such as ease of retrieval—when making judgments (Lipko et al. 2012; van Loon et al. 2017). Interleaved learning may help calibrate these judgments by making the learning process more effortful and less fluent. van Loon et al. (2017) found that when children learned the definitions of concepts, they tended to overestimate their performance on subsequent tests. In their study, children in grades 3 through 6 (ages 8–11) were asked to study definitions such as “Starboard—Starboard is the right side of a ship. The captain turns the ship to starboard.” Later, during the test phase, they were shown the term “Starboard” and asked to write its definition. Results showed that children significantly overestimated their future test performance, and this overestimation decreased with age. van Loon et al. (2017) suggested that such miscalibration is partly due to children’s limited use of accessibility cues when making judgments. Similarly, Pillow and Pearson (2015) pointed out that 9-year-olds often fail to incorporate cues related to cognitive effort when evaluating their own learning. Interleaved learning, which is known to increase the perceived level of cognitive effort (Janssen et al. 2023; Kirk-Johnson et al. 2019), may provide children with more salient internal cues about task difficulty and their own understanding. By encouraging greater awareness of effortful processing, interleaved learning might help children make more accurate self-assessments of their performance. To our knowledge, it remains unknown whether interleaved learning (vs. blocked learning) facilitates children’s metacognitive judgment of learning. Building on this idea, the present study aims to examine whether interleaved learning improves the accuracy of children’s metacognitive judgments in the context of natural rock category learning.

## 2. Overview of the Current Study

Building on the above analysis, several open questions remain regarding the effects of interleaved learning. First, it is unclear whether children can benefit from interleaved learning in the same way that young adults do when learning natural rock categories. Second, it is important to examine whether interleaved learning can enhance metacognitive accuracy—specifically, the accuracy of learners’ judgments about their own category learning performance.

To address these questions, the present study examines two key issues: (1) whether interleaved learning enhances children’s categorization accuracy in natural rock category learning, as it does for young adults; and (2) how interleaved learning affects the accuracy of category learning judgments in both children and young adults. We focused on elementary school children aged 9, who studied natural rock categories and were subsequently asked to identify novel category members. Middle childhood is an important developmental stage for metacognitive judgment ability (Dong et al. 2022; van Loon et al. 2017), during which children’s differential sensitivity patterns are still developing compared to adults (Koriat et al. 2014). Thus, it provides a promising window for investigating category learning judgments in the context of natural category learning.

In Experiment 1, we examined how example sequences (interleaved vs. blocked) influenced both classification accuracy and the accuracy of category learning judgments in natural rock category learning among children and young adults. After completing each learning condition, participants made global judgments of their overall category learning. In this experiment, learning time was experimenter-controlled.

To further confirm and extend the findings from Experiment 1, Experiment 2 introduced a key modification: self-paced example learning time. Previous research with adults has shown that self-paced learning can enhance learning performance by allowing learners to regulate their study time based on their perceived understanding and task difficulty (Dunlosky and Ariel 2011). By enabling learners to manage cognitive load more effectively without feeling rushed, self-paced learning may support better comprehension and retention during category learning. Furthermore, some studies suggest that self-paced learning places greater demands on executive control (Souchay and Isingrini 2004). Prior research has also shown that individual differences in executive functioning can predict the extent to which learners benefit from interleaved versus blocked learning formats (e.g., Park et al. 2023). Taken together, these findings suggest that under self-paced learning conditions, interleaved learning may pose greater challenges than blocked learning—particularly for learners with developing executive skills—potentially limiting the extent to which they can benefit from it. Therefore, Experiment 2 adopted a self-paced learning paradigm based on Wahlheim et al. (2011), allowing participants to control how long to study each example and when to move on to the next. Participants again made global category learning judgments after each learning condition. Additionally, during the learning phase, they were asked to predict their ability to correctly classify novel examples in the upcoming test, providing a direct, ongoing measure of their metacognitive monitoring. Previous research suggests that judgments made during learning (e.g., item-by-item judgments) are often more sensitive to actual learning performance than global post-learning judgments (e.g., Hacker et al. 2008). Building on this idea, Experiment 2 explored how learning sequences influence both classification accuracy and the accuracy of metacognitive judgments under self-paced learning conditions.

## 3. Experiment 1

### 3.1. Method

#### 3.1.1. Participants

To ensure adequate statistical power, we conducted a sensitivity analysis using G*Power 3.1. The results indicated that our final sample of 52 participants (26 children: *M* = 9.53 years, *SD* = 0.51, 15 girls; 26 young adults: *M* = 18.88 years, *SD* = 0.95, 20 females) could detect effect size 1.16 with 95% power for the 2 × 2 mixed design. The children were recruited from local elementary schools in Guangzhou, China. They received a small gift (sticky notes) for participation, and their parents provided written informed consent. The young adults were undergraduate students from a large university in Guangzhou, who participated in exchange for partial course credit. The experiment was conducted in December 2024 and approved by the Ethics Committee of the College of Education at Guangzhou University.

#### 3.1.2. Design

This study used a 2 (presentation sequence: blocked vs. interleaved, within-subjects) × 2 (age group: children vs. young adults, between-subjects) mixed design.

#### 3.1.3. Materials

The natural category stimuli consisted of 60 images of rocks drawn from the set developed by Nosofsky et al. (2018). These images represented six rock types, Anthracite, Basalt, Breccia, Conglomerate, Gabbro, and Obsidian, with ten exemplars per type. For each participant, three of the six rock types were randomly assigned to the blocked learning condition, and the other three to the interleaved learning condition. The specific assignment of rock types to learning conditions was randomized across participants. For each rock type, six of the ten images were randomly selected for use in the learning phase, and the remaining four images were reserved for the test phase.

#### 3.1.4. Procedure

The experiment consisted of two parts, each involving the learning and testing of three different rock categories, for a total of six categories. Three categories were randomly assigned to the first part and the remaining three to the second part. The order in which participants experienced the two learning conditions—blocked and interleaved—was counterbalanced across participants (i.e., Blocked–Interleaved or Interleaved–Blocked).

**Figure 1:**
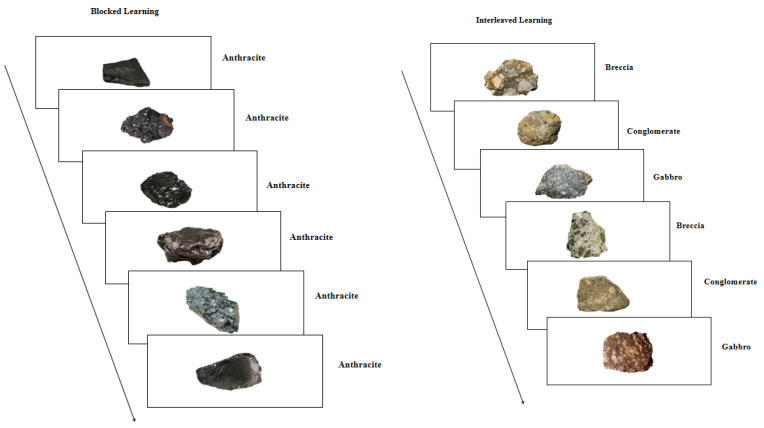
Example sequences for blocked and interleaved learning conditions. Rock pictures were presented in either a blocked or interleaved sequence using a within-subjects design. In the blocked condition, all six rock pictures from the same rock type were shown in random order. In the interleaved condition, one rock picture from each of the three interleaved rock types was shown in random order. For each participant, pictures from three rock types were studied in a blocked sequence, while pictures from the other three rock types were studied in an interleaved sequence. The sequence was counterbalanced across participants, with half beginning with a blocked sequence (BBB III) and the other half with an interleaved sequence (III BBB).

In the blocked condition, participants studied six consecutive exemplars from Category A, followed by six from Category B, and then six from Category C. In the interleaved condition, exemplars from the three categories were presented in an alternating fashion across trials (e.g., A1, B1, C1, A2, B2, C2), continuing until all 18 exemplars (six per category) had been shown (see Figure 1). Regardless of condition, participants were instructed to actively learn the categories with the expectation of classifying new exemplars in a later test. On each trial, a rock image was presented along with three clickable buttons, each labeled with a possible rock type. Participants were asked to infer the category of the rock and select their response by clicking the corresponding button. Participants were allowed to respond at any time within the 4-s trial window; however, each trial had a maximum duration of 4 s, after which it automatically advanced to the feedback phase. If a response was made within this window, feedback indicating whether the response was “Correct” or “Incorrect” was displayed for 2 s. If no response was made, feedback indicated that the response was “Incorrect.” Regardless of response accuracy, the correct rock type was always presented alongside the image. Each trial thus lasted a total of 6 s. Each rock image appeared only once in its designated sequence (blocked/interleaved) and was not reused in later test phases.

After completing the learning phase in the first part of the experiment, participants were asked to make a judgment of learning—specifically, to rate how confident they were in their ability to accurately classify new images from the learned categories in the upcoming test. Next, participants completed a distractor task, which involved performing a mental arithmetic task (subtracting 3 repeatedly from 150). Following the distractor, a categorization test was administered. Participants were shown 12 new rock images—four exemplars from each of the three learned categories—in a random order. No time limit was imposed for responses, and no feedback was provided during this phase. After each response, the next image was presented immediately.

The second part of the experiment followed the same procedure as the first but involved the remaining three rock categories, which were learned under either the interleaved or blocked learning condition, depending on the counterbalancing scheme.

### 3.2. Results and Discussion

**Figure 2:**
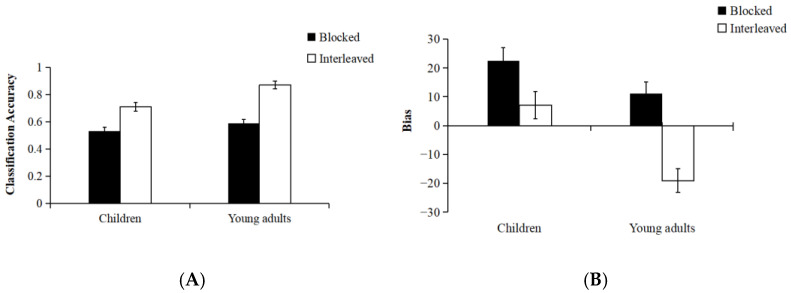
Final classification test performance and category learning judgments bias as a function of presentation sequence (interleaving vs. blocking) across children and young adults in Experiment 1. Panels (**A**,**B**) display the classification test performance and category learning judgments bias for children and young adults, respectively. Error bars represent one standard error of the mean.

***Classification accuracy.*** See Table 1 for descriptive statistics. A mixed ANOVA was conducted with age group (children vs. young adults) as a between-subjects factor and presentation sequence (interleaved vs. blocked) as a within-subjects factor (see Figure 2A). A Bonferroni correction was applied to all pairwise comparisons to maintain a family-wise Type I error rate of 0.05. Classification accuracy was higher in the interleaved condition compared to the blocked condition [*F*(1, 50) = 91.56, *p* < 0.01, η^2^_p_ = 0.65]. In addition, young adults demonstrated significantly higher classification accuracy than children [*F*(1, 50) = 11.81, *p* < 0.01, η^2^_p_ = 0.19]. A significant interaction between presentation sequence and age group was found [*F*(1, 50) = 4.80, *p* = 0.03, η^2^_p_ = 0.09]. Further simple effects analyses revealed that classification accuracy was significantly higher in the interleaved condition than in the blocked condition for both children and young adults [children: *F*(1, 50) = 27.21, *p* < 0.01, η^2^_p_ = 0.35; young adults: *F*(1, 50) = 69.15, *p* < 0.01, η^2^_p_ = 0.58]. The interaction effect reflected a greater difference in classification accuracy between the interleaved and blocked conditions in young adults compared to children, *t*(50) = 2.19, *p* = 0.03, Cohen’s *d* = 0.61. These results suggest that the interleaved learning sequence facilitates categorization accuracy across both age groups, with young adults benefiting more from interleaving than children. This finding highlights potential age-related differences in the effectiveness of interleaved learning.

***Category Learning Judgments magnitude***. A mixed ANOVA was conducted to examine judgment magnitude, with age group (children vs. young adults) as a between-subjects factor and presentation sequence (interleaved vs. blocked) as a within-subjects factor (see Table 1). There was no significant main effect of age group [*F*(1, 50) = 2.05, *p* = 0.16, η^2^_p_ = 0.04], nor was there a significant main effect of presentation sequence [*F*(1, 50) = 0.17, *p* = 0.68, η^2^_p_ < 0.01]. Additionally, the interaction between age group and presentation sequence was not significant [*F*(1, 50) = 2.05, *p* = 0.16, η^2^_p_ = 0.04].

***Category Learning Judgments bias.*** Bias was calculated as the difference between a participant’s CLJ and their classification accuracy (multiplied by 100), with positive values reflecting overestimation and negative scores reflecting underestimation. Mean bias is presented in Table 1. A Bonferroni correction was applied for all pairwise comparisons to maintain a family-wise Type I error rate of 0.05. A 2 (age group: children vs. young adults) × 2 (presentation sequence: interleaved vs. blocked) mixed ANOVA revealed that bias was greater in the blocked condition than in the interleaved condition [see Figure 2B, *F*(1, 50) = 48.65, *p* < 0.01, η^2^_p_ = 0.49]. Using 0 as the accuracy threshold, the blocked condition showed significant overestimation, while the interleaved condition’s judgments were accurately aligned with 0 [blocked condition: *t*(25) = 6.11, *p* < 0.01, Cohen’s *d* = 1.20; interleaved condition: *t*(25) = −1.85, *p* = 0.08, Cohen’s *d* = −0.36]. The main effect of age group was significant, *F*(1, 50) = 12.38, *p* < 0.01, η^2^_p_ = 0.20, monitoring bias was significantly higher in children than in young adults. Specifically, mean bias exceeded zero for children, *t*(25) = 3.60, *p* < 0.01, Cohen’s *d* = 0.71. There was no difference between monitoring bias and 0 for young adults, *t*(25) = −1.16, *p* = 0.26, Cohen’s *d* = −0.22. This implies that children overestimate their categorization performance whereas young adults accurately assess their categorization performance.

Furthermore, there was a significant interaction between age group and presentation sequence, *F*(1, 50) = 6.25, *p* = 0.02, η^2^_p_ = 0.11. Simple effects analyses further revealed no significant difference in monitoring bias between children and young adults in the blocked condition, *F*(1, 50) = 2.73, *p* = 0.11, η^2^_p_ = 0.05. Both children and young adults exhibited monitoring bias scores significantly greater than zero [children: *t*(25) = 4.13, *p* < 0.01, Cohen’s *d* = 0.81; young adults: *t*(25) = 2.74, *p* = 0.01, Cohen’s *d* = 0.54]. This indicates that, under the blocked condition, both age groups overestimated their categorization accuracy.

However, in the interleaved condition, monitoring bias was significantly higher among young adults than children, *F*(1, 50) = 21.62, *p* < 0.01, η^2^_p_ = 0.30. Further analyses indicated that children’s monitoring bias did not significantly differ from zero, whereas young adults significantly underestimated their categorization performance [children: *t*(25) = 1.68, *p* = 0.11, Cohen’s *d* = 0.33; young adults: *t*(25) = −5.66, *p* < 0.01, Cohen’s *d* = −1.11]. This suggests that children accurately assessed their categorical performance, whereas young adults underestimated their own performance under the interleaved condition.

Experiment 1 extends the findings of previous research on the advantages of interleaved learning (Babineau et al. 2022; Birnbaum et al. 2013; Do and Thomas 2023; Kornell and Bjork 2008; Kang and Pashler 2012; Yan and Sana 2021; Zulkiply and Burt 2013), specifically examining its impact on children. The results from Experiment 1 demonstrated that interleaved learning enhanced classification accuracy for both children and young adults. Moreover, we found that this advantage was more pronounced in young adults compared to children. Furthermore, regarding judgment bias in category learning, both groups exhibited overestimation under the blocked condition. However, under the interleaved condition, young adults showed a stronger tendency to underestimate their performance. In contrast, children’s self-assessments remained positively biased, but were more accurate than in the blocked condition. This pattern suggests age-related differences in how interleaved learning affects natural category learning.

## 4. Experiment 2

In the present experiment, we aimed to replicate and extend the findings from Experiment 1 in a self-paced learning context. Unlike in Experiment 1, where study time was fixed and externally controlled, participants in Experiment 2 were allowed to determine how long to study each example and when to proceed (Wahlheim et al. 2011). While self-paced learning may reduce time pressure and allow for more flexible study (Dunlosky and Ariel 2011), it also requires learners to monitor their understanding and regulate their study behavior—skills that depend on executive functions (Souchay and Isingrini 2004). Moreover, previous research has shown that executive functioning can influence the extent to which learners benefit from blocked versus interleaved study formats (Park et al. 2023), highlighting the importance of considering self-paced conditions when evaluating these strategies. Therefore, Experiment 2 examined whether the learning benefits observed in Experiment 1 would generalize to a self-paced context, and whether blocked and interleaved study formats would differentially affect learning across age groups under these conditions.

### 4.1. Method

#### 4.1.1. Participants and Design

There were 76 participants in Experiment 2 (37 children: *M* = 9.22 years, *SD* = 0.42, 15 girls; 39 young adults: *M* = 20.74 years, *SD* = 2.19, 20 females). A sensitivity analysis using G*Power 3.1 showed that the final sample (N = 76) was sufficient to detect an effect size of 1.03 with 95% power in a 2 × 2 mixed-design ANOVA. Experiment 2 followed the same procedure as Experiment 1 in terms of participant recruitment, ethical approval, and experimental design. Specifically, a 2 (presentation sequence: blocked vs. interleaved, within-subjects) × 2 (age group: children vs. young adults, between-subjects) mixed design was used.

#### 4.1.2. Materials and Procedure

**Figure 3:**
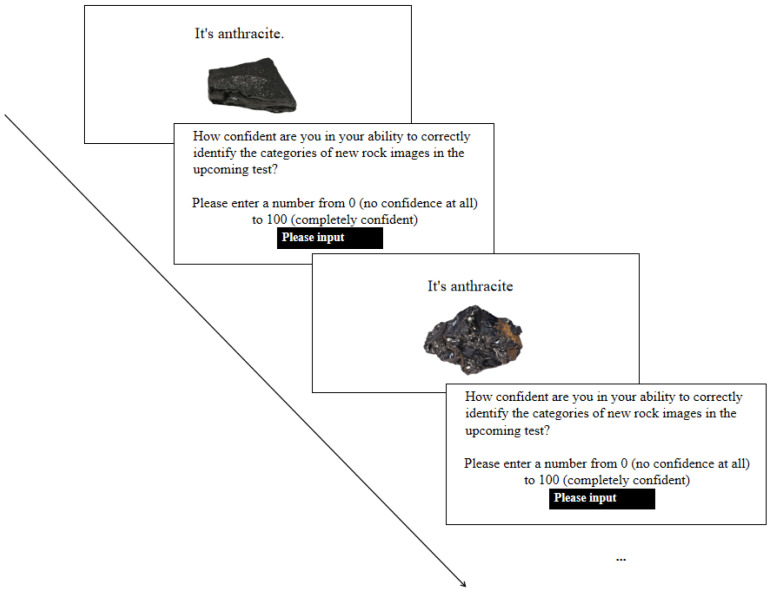
Illustration of the study phase in Experiment 2. Participants self-paced the presentation of each rock image and made a learning judgment immediately after studying each item. For each participant, pictures from three rock types were studied in a blocked sequence, and those from the remaining three types were studied in an interleaved sequence. The sequence was counterbalanced across participants, with half beginning with a blocked sequence (BBB III) and the other half with an interleaved sequence (III BBB).

The learning materials used in Experiment 2 were identical to those in Experiment 1. However, unlike in Experiment 1, the learning judgments in Experiment 2 was made immediately after each item was studied, rather than at a later stage. In addition, the timing of each rock picture’s presentation was self-paced and determined by the participants. Specifically, during the learning phase, participants were presented with pictures of rocks along with the corresponding rock type names. They could autonomously control the duration of time each rock picture was displayed. After completing the study of each rock picture, participants were asked to make a learning judgment (see Figure 3). All other aspects of the procedure remained the same as in Experiment 2.

### 4.2. Results and Discussion

**Figure 4:**
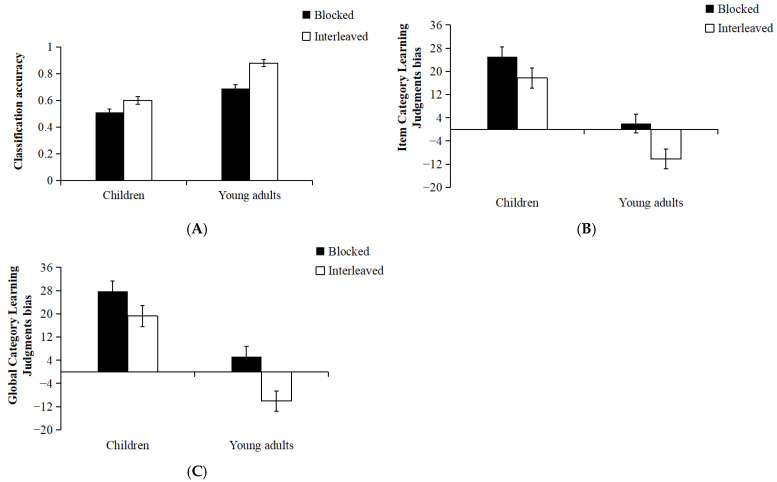
Final classification test performance and category learning judgments bias as a function of presentation sequence (interleaving vs. blocking) across children and young adults in Experiment 2. Panels (**A**–**C**) show classification test performance, item-level category learning judgments bias, and global category learning judgments bias for children and young adults, respectively. Error bars represent ±1 standard error of the mean.

***Classification accuracy***. The average performance for each condition on the classification accuracy is presented in Figure 4A and Table 2. A 2 × 2 mixed ANOVA was conducted, with Sequence (Blocked, Interleaved) as a within-subjects factor and Age Group (Children, Young Adults) as a between-subjects factor. To control the family-wise Type I error rate at 0.05, a Bonferroni correction was applied to all pairwise comparisons. The analysis revealed a significant main effect of sequence: classification accuracy was higher in the interleaved condition than in the blocked condition, *F*(1, 74) = 29.95, *p* < 0.01, η^2^_p_ = 0.29. There was also a main effect of age group, *F*(1, 74) = 58.07, *p* < 0.01, η^2^_p_ = 0.44; young adults’ classification accuracy was higher than that of children. Importantly, there was a significant Sequence × Age Group interaction, *F*(1, 74) = 3.90, *p* = 0.05, η^2^_p_ = 0.05. Further simple effects analyses showed that for children, classification accuracy was significantly higher in the interleaved condition than in the blocked condition, *F*(1, 74) = 6.10, *p* = 0.02, η^2^_p_ = 0.08. However, for young adults, classification accuracy was significantly higher in the interleaved condition than in the blocked condition, *F*(1, 74) = 28.49, *p* < 0.01, η^2^_p_ = 0.28. The interaction manifested as a more pronounced difference between interleaved and blocked learning in young people (*M* = 0.19, *SD* = 0.17) compared to children (*M* = 0.09, *SD* = 0.25), *t*(74) = 1.98, *p* < 0.05, Cohen’s *d* = 0.45. These results suggest that the benefit of interleaved learning in classification accuracy is clearly more pronounced in young adults than in children.

***Item Category Learning Judgments magnitude***. The average performance for each condition on the category learning judgments magnitude is presented in Table 2. A 2 (Sequence: Blocked, Interleaved) × 2 (Age Group: Children, Young Adults) mixed-design ANOVA revealed a significant main effect of sequence, F(1, 74) = 13.75, *p* < 0.01, η^2^_p_ = 0.16. Participants made significantly higher judgments in the interleaved condition than in the blocked condition. A significant Sequence × Age Group interaction was observed, *F*(1, 74) = 8.07, *p* < 0.01, η^2^_p_ = 0.10. Further simple effects analyses revealed that, for children, the magnitude of judgments did not significantly differ between the interleaved and blocked conditions. In contrast, young adults showed significantly higher judgment magnitude in the interleaved condition than in the blocked condition [for children: *F*(1, 74) = 0.37, *p* = 0.55, η^2^_p_ < 0.01; for young adults: *F*(1, 74) = 22.00, *p* < 0.01, η^2^_p_ = 0.23].

***Item Category Learning Judgments bias***. The average monitoring bias scores for each condition are presented in Figure 4B and Table 2. A 2 (Sequence: Interleaved vs. Blocked; within-subject) × 2 (Age Group: Children vs. Young Adults; between-subject) mixed-design ANOVA revealed a significant main effect of learning sequence, *F*(1, 74) = 13.21, *p* < 0.01, η^2^_p_ = 0.15. Monitoring bias was significantly lower in the interleaved condition than in the blocked condition. Specifically, under the blocked learning condition, participants’ monitoring bias was significantly greater than zero, indicating an overestimation of their classification accuracy. In contrast, under the interleaved learning condition, monitoring bias did not significantly differ from zero, suggesting that participants were more accurate in assessing their future test performance [blocked learning condition: *t*(74) = 5.00, *p* < 0.01, Cohen’s *d* = 0.57; interleaved learning: *t*(74) = 1.07, *p* > 0.05, Cohen’s *d* = 0.12].

The main effect of age group was significant, *F*(1, 74) = 43.54, *p* < 0.01, η^2^_p_ = 0.37. Monitoring bias was significantly higher in children than in young adults. For children, monitoring bias was significantly greater than zero, suggesting that they overestimated their future classification accuracy. In contrast, young adults’ monitoring bias did not significantly differ from zero, indicating that they accurately assessed their future performance [children: *t*(36) = 7.62, *p* < 0.01, Cohen’s *d* = 1.25; young adults: *t*(38) = −1.57, *p* = 0.13, Cohen’s *d* = −0.25 ]. The interaction between learning sequence and age group was not significant, *F*(1, 74) = 0.60, *p* = 0.44, η^2^_p_ < 0.01.

Although interleaving reduced monitoring bias values for both children and young people compared to blocked learning (see Figure 4B), the effects on the two groups still diverged. Specifically, interleaving led to greater underestimation of test performance in young people [compared to zero: blocked learning, *t*(38) = 0.63, *p* = 0.53, Cohen’s *d* = 0.10; interleaved learning, *t*(38) = −3.56, *p* < 0.01, Cohen’s *d* = −0.57]. In contrast, for children, interleaved learning reduced their overestimation of performance relative to blocked learning [compared to zero: blocked learning, *t*(36) = 7.52, *p* < 0.01, Cohen’s *d* = 1.24; interleaved learning, *t*(36) = 4.34, *p* < 0.01, Cohen’s *d* = 0.71].

***Global Category Learning Judgments magnitude***. The average magnitude of global category learning judgments for each condition is presented in Table 2. A 2 (Sequence: Blocked, Interleaved; within-subjects) × 2 (Age Group: Children, Young Adults; between-subjects) mixed-design ANOVA was conducted. The analysis yielded no significant main effect of age group, *F*(1, 74) = 1.48, *p* = 0.23, η^2^_p_ = 0.02, nor of sequence, *F*(1, 74) = 2.13, *p* = 0.15, η^2^_p_ = 0.03. The interaction between age group and sequence was also non-significant, *F*(1, 74) = 1.61, *p* = 0.21, η^2^_p_ = 0.02.

***Global Category Learning Judgments bias***. Figure 4C and Table 2 present the average bias scores (i.e., the difference between participants’ category learning judgments and actual accuracy) across conditions. A 2 (Exemplar Sequence: Interleaved vs. Blocked) × 2 (Age Group: Children vs. Young Adults) mixed-design ANOVA was conducted on participants’ global category learning judgments bias scores. The results revealed a significant main effect of age group, *F*(1, 74) = 39.39, *p* < 0.01, η^2^_p_ = 0.35, with significantly lower bias scores in young adults than in children. To examine whether participants systematically over- or underestimated their performance, one-sample *t*-tests were conducted for each age group. The results showed that children’s bias scores were significantly greater than zero, indicating a tendency to overestimate their future performance. In contrast, young adults’ bias scores did not significantly differ from zero, suggesting more accurate self-assessments [children: *t*(36) = 7.20, *p* < 0.01, Cohen’s *d* = 1.20; young adults: *t*(38) = −0.93, *p* = 0.36, Cohen’s *d* = −0.15]. The interaction was not significant, *F*(1, 74) = 18.73, *p* < 0.01, η^2^_p_ = 0.20.

The analysis revealed a significant main effect of exemplar sequence, *F*(1, 74) = 1.49, *p* = 0.23, η^2^_p_ = 0.02. Participants exhibited significantly lower bias in the interleaved condition compared to the blocked condition. In the blocked condition, participants significantly overestimated their classification accuracy; In the interleaved condition, bias scores did not differ significantly from zero [blocked condition: *t*(75) = 5.84, *p* < 0.01, Cohen’s *d* = 0.67; interleaved condition: *t*(75) = 1.39, *p* = 0.17, Cohen’s *d* = 0.16]. Among children, bias scores were significantly greater than zero in both the blocked and interleaved conditions, indicating overestimation across both learning sequences [blocked condition: *t*(36) = 7.42, *p* < 0.01, Cohen’s *d* = 1.22; interleaved condition: *t*(36) = 4.61, *p* < 0.01, Cohen’s *d* = 1.19]. Notably, interleaving significantly reduced children’s bias compared to blocking (*p* = 0.03). Among young adults, bias scores did not significantly differ from zero in the blocked condition, but were significantly below zero in the interleaved condition, indicating a tendency to underestimate performance [blocked condition: *t*(38) = 1.61, *p* = 0.12, Cohen’s *d* = 0.76; interleaved condition: *t*(38) = −3.57, *p* < 0.01, Cohen’s *d* = −0.57]. Notably, although interleaved learning reduced overall bias compared to blocked learning (*p* < 0.01), it resulted in underestimation among young adults. These findings suggest that interleaved learning leads to reduced judgment bias relative to blocked learning, particularly among children. However, for young adults, interleaving induced underestimation of performance.

In Experiment 2, we employed a self-paced learning scenario and found that interleaved learning improved categorization accuracy for both children and young adults, although the effect sizes were smaller compared to Experiment 1. Specifically, the effect size for children was 0.08 (compared to 0.35 in Experiment 1), and for young adults, it was 0.28 (compared to 0.58 in Experiment 1). These findings replicate the core pattern observed in Experiment 1, suggesting that the interleaving benefit generalizes to self-paced learning, albeit with reduced magnitude. Moreover, interleaved learning produced age-related differences in category learning judgments. Young adults showed a stronger interleaving benefit than children in terms of judgment bias, as reflected in both item-by-item and global category learning judgments. Interestingly, interleaved learning appeared to reduce children’s tendency to overestimate their performance, whereas it led to underestimation in young adults. These results suggest that interleaving not only influences actual learning outcomes but also modulates metacognitive evaluations in distinct ways across age groups.

## 5. General Discussion

A substantial body of research has shown that interleaved learning facilitates young adults’ acquisition of highly confusable categories (Birnbaum et al. 2013; Do and Thomas 2023; Kornell and Bjork 2008; Kang and Pashler 2012; Yan and Sana 2021; Zulkiply and Burt 2013). Building on this work, we investigated how exemplar sequencing—specifically interleaving versus blocking—affects natural category learning in both children and young adults. Importantly, our study not only examined categorization accuracy but also assessed the accuracy of participants’ category learning judgments. While Experiment 1 employed an experimenter-paced procedure with predetermined study durations, Experiment 2 introduced a self-paced learning condition in which participants controlled the amount of time spent on each example.

We found that interleaved learning promoted categorization accuracy more effectively than blocked learning for both children and young adults, consistent with previous studies (Brunmair and Richter 2019; Do and Thomas 2023). Moreover, young adults benefitted significantly more from interleaved learning than did children. In terms of category learning judgment bias, interleaving reduced children’s overestimation but increased underestimation in young adults. Together, these findings suggest that the sequencing of exemplars influenced not only learning outcomes but also learners’ metacognitive awareness of their own learning.

### 5.1. The Impact of Exemplar Sequence on Natural Category Categorization in Children and Young Adults

Across two experiments, we demonstrated that interleaved learning, relative to blocked learning, facilitates natural category learning in both children and young adults. Interleaved study consistently led to better performance on the final classification test, particularly for categories that were more easily learned. These findings are in line with previous research (Do and Thomas 2023) and meta-analytic evidence (Brunmair and Richter 2019) supporting the benefits of interleaved learning for visual category acquisition. Importantly, our study contributes to the existing literature by providing evidence of an interleaving effect in children. While most prior research on the benefits of interleaved learning for highly confusable categories has concentrated on young adults (Brunmair and Richter 2019), evidence for younger learners remains scarce. The current study replicates the positive effects of interleaving across a broader age range—including children as young as 9 years old—highlighting the generalizability of these benefits for natural category learning in both children and young adults.

Furthermore, across Experiments 1 and 2, we observed that young adults benefited more from interleaved learning than did children. In Experiment 1, where learning time was fixed by the experimenter, interleaved learning demonstrated a clear advantage over blocked learning for both children (effect size = 0.35) and young adults (effect size = 0.58). However, when participants regulated their own study time in Experiment 2, the advantage of interleaved learning was reduced—though it still remained (children: 0.08; young adults: 0.28). Previous research has shown that executive function abilities predict the extent to which learners benefit from interleaved compared to blocked sequence (Park et al. 2023). In self-paced learning contexts, learners are required to monitor their understanding and determine when to proceed, which tends to engage executive functions such as working memory and inhibitory control (Souchay and Isingrini 2004). These additional demands may make it more difficult for learners to simultaneously manage their study time and attend to the critical contrasts between categories.

Moreover, the age-related differences observed in our study further support this interpretation, suggesting that developmental differences in executive functions may contribute to the effectiveness of interleaved learning. One key component of executive functions is selective attention (Morea and Calvete 2021), which enables learners to focus on relevant information and suppress distractions during learning. The greater benefits observed in young adults may reflect their more advanced attentional control abilities. As attentional regulation matures from childhood into young adulthood, young adults may be better equipped to capitalize on the discriminative contrasts emphasized by interleaved learning. According to the discriminative-contrast hypothesis, the benefit of interleaving—particularly for high-similarity categories—arises from the opportunity to contrast successive examples from different categories, which facilitates the identification of key distinguishing features (Chen et al. 2021; Kornell and Bjork 2008; Kang and Pashler 2012). When examples are alternated across categories, learners must selectively attend to diagnostic features presented in close succession (Nemeth et al. 2021). Previous research suggests that young adults, compared to children, are more proficient at directing attention toward category-relevant information (Deng and Sloutsky 2016; Frank et al. 2021). Therefore, the greater benefit observed in young adults may stem from their more advanced selective attention abilities, allowing them to better exploit the opportunities provided by interleaved learning. Taken together, these findings suggest a potential role for individual differences in cognitive resources—especially attentional control—in shaping the effectiveness of interleaved learning. Further research is needed to test this possibility directly.

### 5.2. The Effect of Exemplar Sequence and Age Group on Category Learning Judgments

Across two experiments assessing both global and item-level category learning judgments in the context of natural category learning, we found that young adults generally made accurate judgments, whereas 9-year-old children tended to overestimate their learning performance. This finding aligns with previous research in the memory domain, which has shown that elementary school children often display overconfidence in their metacognitive judgments (Roebers 2002, 2014; Schneider 2015). Notably, such overconfidence tends to decline with age, with significant developmental changes typically occurring between 8 and 12 years (van Loon et al. 2017). For example, 10-year-olds have been found to show less overestimation than 8-year-olds (van Loon et al. 2017). This developmental improvement has been attributed to older children’s increased reliance on accessibility-based cues—such as how easily information comes to mind—which enhances their monitoring accuracy (van Loon et al. 2017). Indeed, research suggests that by age 11, children begin to show adult-like reliance on accessibility cues in their learning judgments, although this ability is still developing. Our findings extend this developmental trajectory to the domain of natural category learning: while 9-year-old children continued to exhibit substantial overestimation of their categorization performance, young adults were able to more accurately assess their learning. These results corroborate earlier work on young learners’ overconfidence (Lipko-Speed 2013; Roebers 2014; Schneider and Lockl 2008), and further indicate that age-related gains in metacognitive accuracy extend beyond memory domains to concept learning involving highly similar, confusable natural categories.

Much prior research has focused on young adults’ metacognitive judgments regarding the effectiveness of interleaved and blocked learning strategies (Janssen et al. 2023; Kirk-Johnson et al. 2019). However, it remains unclear whether these strategies also affect the accuracy of category learning judgments, particularly in children. To address this gap, we examined how accurately children and young adults judged their category learning after studying natural categories under interleaved and blocked learning conditions. The sequence of category examples influenced judgment accuracy in both age groups, though in different ways: interleaved learning reduced overestimation in children, while it led to greater underestimation in young adults, relative to blocked learning.

These contrasting patterns suggest age-related differences in how learners interpret disfluency as a cue for learning success. As discussed above, young adults are generally able to assess their category learning with a relatively high degree of accuracy. However, they tend to underestimate their performance when learning feels disfluent (Koriat 1997). Interleaved learning, compared to blocked learning, often induces such disfluent experiences (Janssen et al. 2023), which may lead young adults to underappreciate their actual learning gains. In contrast, children’s metacognitive monitoring skills are still developing, and they tend to overestimate their performance across different learning conditions (Finn and Metcalfe 2014; Lipko et al. 2012). Even when they begin to rely on valid cues, they may still exhibit overconfidence in their judgments (van Loon et al. 2017). Notably, our findings suggest that 9-year-old children are sensitive to the disfluency introduced by interleaved learning. Although they continued to overestimate their performance overall, the presence of disfluency appeared to calibrate their judgments, leading to a reduction in overestimation compared to blocked learning.

## 6. Limitations and Suggestions for Future Research

One limitation of the present study is that learning outcomes were assessed immediately following an arithmetic filler task, rather than after a longer retention interval (e.g., one week). As a result, the current design does not allow for strong conclusions about the long-term effects of interleaving. To more comprehensively evaluate the durability of interleaved versus blocked learning across natural category structures, future research should incorporate delayed testing. Prior studies have demonstrated that interleaved learning can enhance long-term retention by promoting better discrimination of critical features across categories (Yan and Schuetze 2022). Incorporating longer delays would help determine whether the performance benefits observed here extend to more enduring forms of learning and thus inform educational applications more reliably. In addition, although the current study provides evidence for the benefits of interleaved learning across age groups, it was conducted in a controlled laboratory setting using abstract category materials. Therefore, its ecological validity remains limited. To evaluate the practical utility of interleaved learning in educational settings, future studies should employ curriculum-based materials and examine learning over extended timescales that are relevant to real classroom instruction.

## 7. Practical Implications for Education

Our findings offer meaningful implications for educational practice. Interleaved learning promoted category learning accuracy in both children and young adults, with greater benefits observed in the latter group. This suggests that interleaved structures can be effectively applied across age groups to enhance conceptual understanding, especially when distinguishing among similar categories. However, interleaving had distinct effects on learners’ metacognitive judgments. For children, it helped reduce overestimation, suggesting that disfluent learning experiences can support more realistic self-assessments during development. For young adults, by contrast, interleaving led to increased underestimation, highlighting the need for instructional support to help them interpret disfluency as a potentially beneficial signal.

Together, these results highlight the importance of aligning learning strategies with developmental differences—not only in terms of learning outcomes but also in metacognitive accuracy. Educators might pair interleaved practice with feedback or reflective prompts to help learners of different ages accurately monitor their progress and better understand the value of effortful learning.

## 8. Conclusions

The present study provided evidence that interleaved learning improves classification accuracy in natural category learning across age groups, with greater benefits observed in young adults. Importantly, it also revealed that study sequence affects metacognitive monitoring in distinct ways: interleaving reduced overconfidence in children but increased underconfidence in young adults. These findings not only confirm the cognitive benefits of interleaved learning but also uncover a developmental dissociation in how learners monitor their own performance under different study conditions. By integrating learning performance with metacognitive evaluation, our study advances understanding of how interleaved learning operates across developmental stages. It highlights that while interleaving can facilitate category learning, its effects on learners’ confidence and self-monitoring are age-dependent, potentially shaping how learners approach and regulate future learning. These insights contribute to both cognitive and educational psychology by emphasizing the need to tailor instructional sequences based on developmental differences in metacognition. Future studies may build on these findings to explore how training or scaffolding can mitigate under- or overconfidence, optimizing learning outcomes across age groups.

Taken together, our findings reinforce the value of interleaved learning while underscoring the importance of considering metacognitive factors in developmental research. They provide a foundation for designing developmentally informed educational interventions that leverage study sequencing to enhance both learning and self-regulated understanding.

## Figures and Tables

**Table 1:** Mean (±SD) accuracy, confidence judgments, and response bias in blocked vs. interleaved conditions for children and young adults.

	Children		Young Adults	
	Interleaved	Blocked	Interleaved	Blocked
Mean Classification accuracy	0.70 (0.17)	0.53 (0.14)	0.87 (0.11)	0.59 (0.17)
Mean category learning judgments magnitude	78.46 (17.21)	75.00 (22.59)	68.38 (14.35)	70.31 (16.82)
Mean bias of category learning judgments	7.94	22.18 **	−19.05 **	11.08 *

Note. * Mean bias differed from zero, *p* < 0.05; ** Mean bias differed from zero, *p* < 0.01. Negative bias scores indicate underestimation of learning performance.

**Table 2:** Mean (±SD) accuracy, confidence judgment (item- and global- category learning), and response bias in blocked vs. interleaved conditions for children and young adults.

	Children		Young Adults	
	Interleaved	Blocked	Interleaved	Blocked
Classification accuracy	0.60 (0.23)	0.51 (0.17)	0.88 (0.10)	0.69 (0.15)
Item category learning judgments magnitude	77.35 (11.22)	76.51 (12.92)	77.41 (13.96)	70.90 (14.77)
Global category learning judgments magnitude	79.92 (15.43)	79.68 (14.43)	77.62 (14.41)	74.13 (16.08)
Item category learning judgments bias	17.17 **	25.14 **	−10.28 **	2.01
Global category learning judgments bias	19.20 **	27.78 **	−10.07 **	5.23

Note. ** Mean bias differed from zero, *p* < 0.01.

## Data Availability

Data are available in the Open Science Framework at https://osf.io/ts6kd/ (accessed on 19 August 2025).
